# Machine Learning Techniques for the Diagnosis of Schizophrenia Based on Event-Related Potentials

**DOI:** 10.3389/fninf.2022.893788

**Published:** 2022-07-08

**Authors:** Elsa Santos Febles, Marlis Ontivero Ortega, Michell Valdés Sosa, Hichem Sahli

**Affiliations:** ^1^Cuban Neuroscience Center, Havana, Cuba; ^2^Department of Electronics and Informatics (ETRO), Vrije Universiteit Brussel (VUB), Brussels, Belgium; ^3^Department of Data Analysis, Faculty of Psychology and Educational Sciences, Ghent University, Ghent, Belgium; ^4^Interuniversity Microelectronics Centre (IMEC), Leuven, Belgium

**Keywords:** multiple kernel learning, schizophrenia, Boruta, feature selection, event related potential, machine learning

## Abstract

**Antecedent:**

The event-related potential (ERP) components P300 and mismatch negativity (MMN) have been linked to cognitive deficits in patients with schizophrenia. The diagnosis of schizophrenia could be improved by applying machine learning procedures to these objective neurophysiological biomarkers. Several studies have attempted to achieve this goal, but no study has examined Multiple Kernel Learning (MKL) classifiers. This algorithm finds optimally a combination of kernel functions, integrating them in a meaningful manner, and thus could improve diagnosis.

**Objective:**

This study aimed to examine the efficacy of the MKL classifier and the Boruta feature selection method for schizophrenia patients (SZ) and healthy controls (HC) single-subject classification.

**Methods:**

A cohort of 54 SZ and 54 HC participants were studied. Three sets of features related to ERP signals were calculated as follows: peak related features, peak to peak related features, and signal related features. The Boruta algorithm was used to evaluate the impact of feature selection on classification performance. An MKL algorithm was applied to address schizophrenia detection.

**Results:**

A classification accuracy of 83% using the whole dataset, and 86% after applying Boruta feature selection was obtained. The variables that contributed most to the classification were mainly related to the latency and amplitude of the auditory P300 paradigm.

**Conclusion:**

This study showed that MKL can be useful in distinguishing between schizophrenic patients and controls when using ERP measures. Moreover, the use of the Boruta algorithm provides an improvement in classification accuracy and computational cost.

## Introduction

Schizophrenia is a severe and persistent debilitating psychiatric disorder with a prevalence of about 1% of the world population (McGrath et al., 2004). Although psychotic symptoms such as hallucinations and delusions are frequently present, impaired information processing is probably the most common symptom (Javitt et al., 1993). This deficit is reflected mainly by deficits in attention and working memory tasks when compared with healthy controls (Li et al., 2018). The diagnosis of schizophrenia is made by psychiatrists by ascertaining the presence of predefined symptoms (or their precursors) with personal interviews. However, in some cases this diagnosis is unclear, or patients are misdiagnosed with Schizophrenia (Coulter et al., 2019). Thus, finding biomarkers for the prediction of individuals with schizophrenia would be desirable to enable choosing the optimal treatment (pharmacologic or non-pharmacologic). Analysis of the electroencephalogram (EEG) during information processing tasks could provide objective complementary measures to support the subjective human-based decision process (Sabeti et al., 2009; Koukkou et al., 2018).

EEG is a non-invasive and low-cost technique used to measure electrical brain activity along with multiple scalp locations. EEG signals have been widely adopted to study mental disorders, such as dementia, epileptic seizures, cognitive dysfunction, among others, as well as schizophrenia (Loo et al., 2016; Olbrich et al., 2016; Horvath et al., 2018). Electrophysiological data reflects the spontaneous activity of myriad brain parcels, but also can include responses to afferent stimuli (Cong et al., 2015). Event-related potentials (ERPs) are electrical responses that are time-locked to a specific stimulus or event and can be used to assess brain dynamics during information processing in specific tasks (Woodman, 2010). When a subject is presented with a series of standard stimuli, interspersed with infrequent deviant stimuli, the Mismatch Negativity (MMN) (Lee et al., 2017) and the P300 (Li et al., 2018) components are generated. This task is known as the oddball paradigm and is used to study schizophrenia since consistent deficits in the P300 and MNN have been reported in this disease (Bramon et al., 2004; Javitt et al., 2017). Although MMN and P300 are usually produced by an infrequent unexpected event in a sequence of auditory stimuli, P300 can also be obtained with visual stimuli. The MMN is of shorter latency and does not require attention to the stimulus (Näätänen et al., 2004), whereas the P300 is of longer latency and requires attention to the stimulus (Huang et al., 2015).

Several studies have reported significant differences in the latency and amplitude of MMN and P300 between controls and patients, suggesting that these features are possible markers of the prodromal phase of schizophrenia (Atkinson et al., 2012; Loo et al., 2016) as well as potential endophenotypes for schizophrenia (Earls et al., 2016). Analysis of a large dataset of auditory P300 ERP (649 controls and 587 patients) confirmed the reliability of this reduced amplitude, with a large effect size (Turetsky et al., 2015). However, these findings of statistically significant differences in a group analysis do not imply that EEG is useful for the prediction of individual schizophrenia cases (Lo et al., 2015), which requires applying a prediction paradigm using Machine Learning.

Machine learning techniques have potential value for assisting the diagnosis of brain disorders (Burgos and Colliot, 2020). Recent works are based on EEG signals for the diagnosis of epilepsy (Tanu, 2018), Alzheimer's disease and dementia (Joshi and Nanavati, 2021), and Parkinson (Maitín et al., 2020), among other disorders. Particularly, ERP measures combined with machine learning techniques are being tested for the classification of schizophrenia. The most common features used are based on the amplitude and latency of different components [e.g., N100 and P300 (Neuhaus et al., 2013), P50 and N100 (Iyer et al., 2012; Neuhaus et al., 2014)], with several classifiers tested. Neuhaus et al. (2013) used visual and auditory oddball paradigms and a k-nearest neighbor (KNN) classifier and obtained a classification accuracy of 72.4 %. The same author with a bigger sample size and a Naive Bayes (NB) classifier achieved a 77.7% of accuracy (Iyer et al., 2012). Laton et al. (2014) evaluated the performance of several classifiers extracting features from auditory/visual P300 and MMN. The results using NB and Decision Tree (without and with AdaBoost) achieved accuracies of about 80%. Recently, Barros et al. (2021) published a critical review that summarizes machine learning-based classification studies to detect SZs based on EEG signals, conducted since 2016. These authors reported that Support Vector Machines (SVM) were the most commonly used classification algorithm, probably due to their computational efficiency. This kernel-based learning method also achieved the best performance in most studies. Nevertheless, to the best of our knowledge, none of the studies focused on ERP for SZ classification have used multiple kernels, employing instead only one specific kernel function.

The multiple kernel learning (MKL) method learns a weighted combination of different kernel functions and can benefit from information coming from multiple sources (Wani and Raza, 2018). A recent survey of artificial intelligence methods for the classification and detection of Schizophrenia (Lai et al., 2021), shows that MKL has been applied to both structural and functional Magnetic Resonance Images (MRI), increasing performance accuracy (Ulaş et al., 2012; Castro et al., 2014; Iwabuchi and Palaniyappan, 2017). Nevertheless, in this review MKL algorithms applied to electrophysiological data have been not reported, although a recent study used EEG dynamic functional connectivity networks to classify SZ based on MKL (Dimitriadis, 2019). To our knowledge, ERP data has not been used to classify SZ using MKL despite its use for other purposes such as brain-computer interfaces (Li et al., 2014; Yoon and Kim, 2017).

Here, we explore the efficacy of MKL for the classification of schizophrenia based on ERP measures extracted from auditory and visual P300 and MMN. Using the same dataset provided by Laton et al. (2014), we extended the set of predictor variables beyond the latency and amplitude of the ERP components, by including additional morphological features (based on time) together with some features extracted from the frequency domain. Due to the huge number of features, the Boruta method (Kursa and Rudnicki, 2010) was applied, which is a wrapper Random Forest (RF) based feature selection algorithm, to estimate the impact of a subset of important and relevant feature variables in the classification accuracy.

## Materials and Methods

### Dataset

The study (Laton et al., 2014) was carried out on data from 54 SZ patients and 54 HC, matched for age and gender. Patients were classified by a semi-structured interview (OPCRIT v4.0) and all participants gave written informed consent. Detailed demographic data can be found in Table 1. EEGs were recorded using a 64-channel and the international 10/10 system, with a sampling frequency of 256 Hz. Three paradigms were used: auditory/visual P300 and MMN. Table 2 shows a brief description of the paradigms and procedures. We refer to Laton et al. (2014) for the study details.

**Table 1:** Demographic data (Laton et al., 2014).

	**Patients**	**Controls**	***P* (*t*-test)**
Number of participants	54	54	
Male	36	36	
Age (years): mean ± std	40.5 ± 10.1	37.6 ± 14.1	0.22
Age (years): range	[22.4, 60.5]	[15.1, 64.4]	
Education (years): mean ± std	12.6 ± 1.80	14.8 ± 2.11	4.84 ×10–5
Disease duration (years): mean ± std	14.8 ± 9.04	–	
Disease duration (years): range	[1, 40]	–	

**Table 2:** Paradigms and procedures (Laton et al., 2014).

	**Auditory P300**	**Visual P300**	
	**Tone**	**Figure**	**Distribution (%)**
Target	1500 Hz 70 dB	Square, side 106 pixels	10
Distractor	500 Hz 70 dB	Circle, diameter 176 pixels	10
Standard	1000 Hz 70 dB	Square, side 158 pixels	80
Inter-stimulus interval was randomized between 1 and 1.5 s. 400 stimuli per test. 100 ms per stimuli. Total test time of 540 s.			
	**MMN**		
	**Tone**	**Duration**	**Distribution**
Duration deviant	1000 Hz 70 dB	250 ms	5%
Frequency deviant	1500 Hz 70 dB	100 ms	5%
Standard	1000 Hz 70 dB	100 ms	90%
Inter-stimulus interval of 300 ms, 1800 tones per test. Total test time of 733 s.			

The signals were filtered using bandpass Butterworth filters with cut-offs at 0.1 and 30 Hz. Epochs were extracted using time windows between −200 and 800 ms for the P300 paradigms (257 discrete data points) and between −100 and 500 ms for the MMN (155 discrete data points). Subsequently, baseline correction, re-referencing to linked ears, and artifact rejection were performed. Finally, epochs were averaged into stimulus-specific responses for each individual, and low-pass filter and baseline correction were re-applied. More details can be found in Laton et al. (2014).

### Feature Extraction

**Figure 1:**
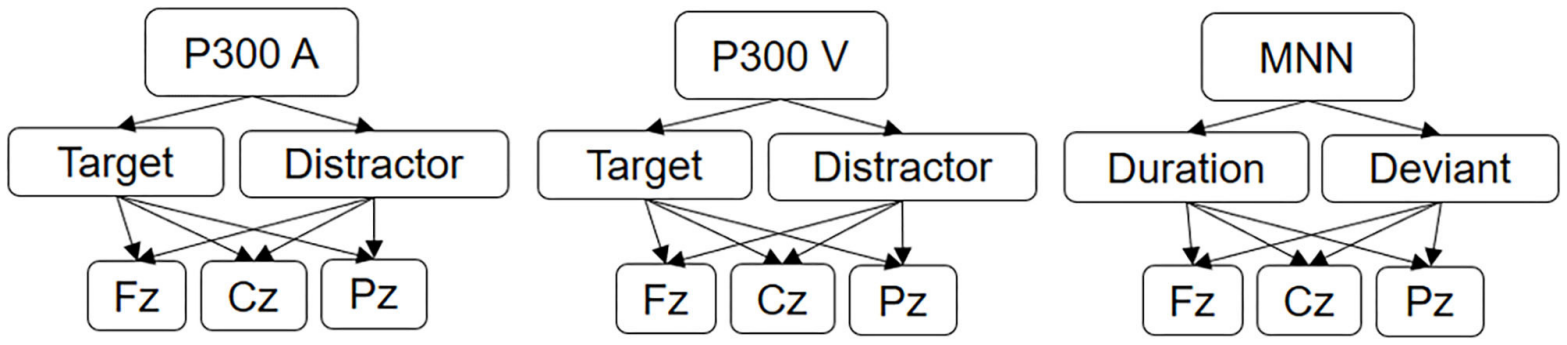
Averaged evoked potential signals used for feature extraction.

The initial set of measurement data consists of averaged signals of 62 channels for each specific response to the three paradigms. This leads to a large amount of data, so it is necessary to transform the initial raw data into a set of features, or signal characteristics that better represent the underlying problem. The process of transforming the signals into numerical features has been carried out on the waveform of ERPs emerged as the averaging of the electrical responses corresponding to the set of stimuli implicated in the detection of rare events (Target and Distractor for P300, Duration and Deviant for MNN), which are more prominent at midline scalp electrode locations Fz, Cz, and Pz (Bénar et al., 2007). As stated, SZ typically exhibits smaller amplitudes in these components compared to HC (Li et al., 2018). Additionally, several studies demonstrated P300 and MMN component differences between SZs and HCs at midline electrodes (Hirayasu et al., 1998; Graber et al., 2019), thus only these channels were considered (see Figure 1).

**Figure 2:**
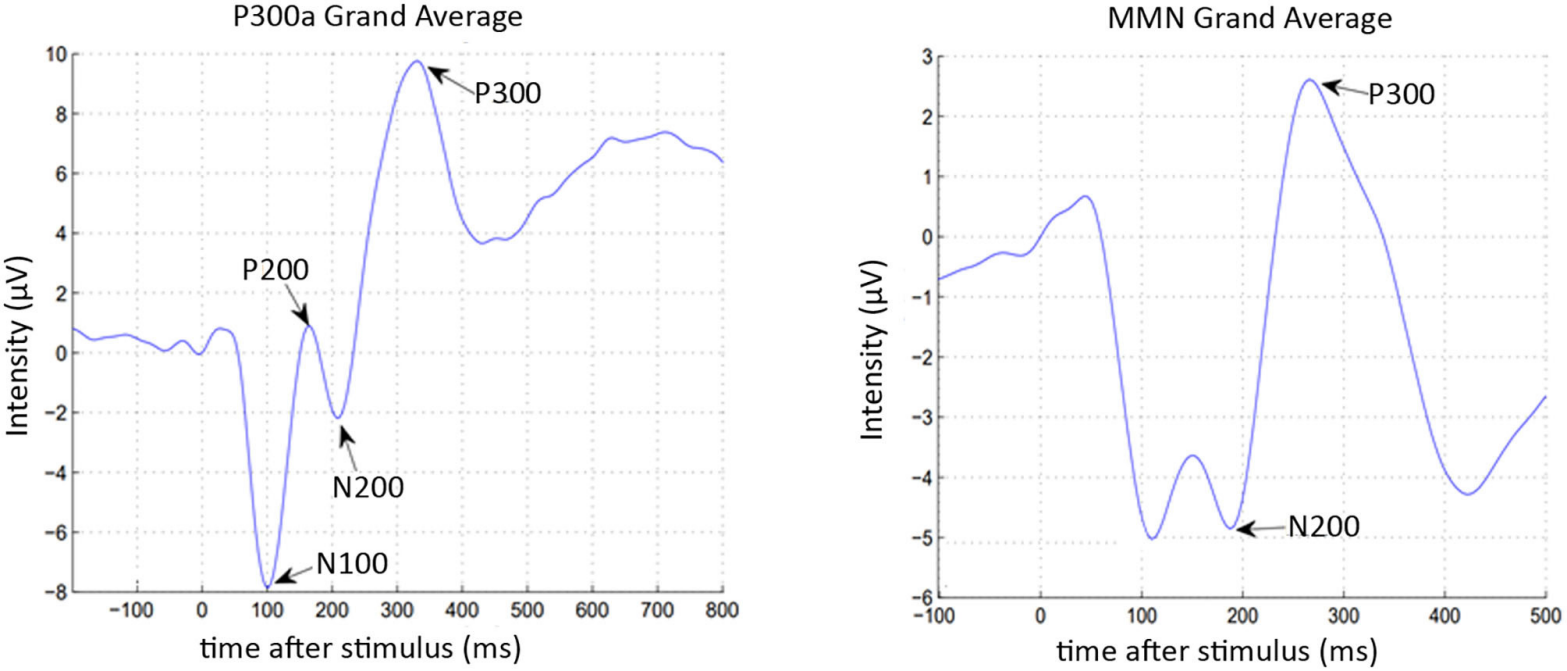
Principal components of P300 tasks (N100, P200, N200, P300) and MMN task (P200, P300).

The set of features can be divided into three categories: peak related features, peak to peak related features, and signal related features. The formal definitions of the used features are given in Annex 1. Some of these features were previously used by other authors to calculate features related to the ERP signal (Kalatzis et al., 2004; Abootalebi et al., 2009). Four peaks for the P300 paradigms (N100, P200, N200, and P300) and two peaks for the MMN paradigm (N200, P300) were considered (see Figure 2). Consequently, the number of features extracted for classification purposes was 726 (282 features for auditory P300 paradigm, 282 for visual P300 paradigm, and 162 for MMN paradigm).

#### Peak Related Features

Peaks were estimated using the same algorithm described in Laton et al. (2014). Four intervals were established around the average latency of the respective peak, measured on the grand averages. The algorithm considered *Amplitude* as the largest absolute value in each interval and *Latency* as the time where the peak appears in the respective time window (interval). The other features were: *Absolute Amplitude, Latency/Amplitude ratio, Absolute Latency/Amplitude ratio, Average Absolute Signal Slope*, and *Slope sign alterations*.

#### Peak to Peak Related Features

Three features were calculated considering the relationship between adjacent selected peaks: the absolute difference between the amplitude of the peak and the next peak in latency order; the difference in latencies of these two peaks; and the slope of the signal in this time window.

#### Signal Related Features

Features considering the area under the curve were calculated: the sum of the positive signal values (*Positive Area*); the sum of the negative signal values (*Negative Area*); the *Total Area*, and *Absolute Total Area*. Two more features related to the whole signal were calculated: the number of times that the amplitude value of the signal crosses the zero y-axis between two adjacent peaks (*Zero Crossing*); and the relation of the number of crosses per time interval (*Zero Cross Density*).

Additionally, frequency domain features were extracted using a Power Spectral Density (PSD) analysis: the frequency with the largest energy content in the signal spectrum (*Mode frequency*) spectrum; the frequency that separates the power spectrum into two equal energy areas (*Median frequency*); and an estimate of the central tendency of the derivate power distributions (*Mean frequency*).

### Feature Scaling

Mapping the feature values of a dataset into the same range is crucial for those algorithms that exploit distances or similarities (Ahsan et al., 2021). The feature values were z-scored, for standardizing them on the same scale by dividing the feature's deviation by the standard deviation in a data set. This improved the numerical stability of the model. Standardization also maintains useful information about outliers and makes the algorithm less sensitive to them (Sahu et al., 2020). The standardized values were then normalized, rescaling them all to values between 0 and 1 using the sigmoid transformation function.

### Feature Selection

After the feature scaling process, feature selection was applied. This is useful for constructing the smallest subset of features from the original set maintaining as much as possible the original meaning of the data. This technique of dimensionality reduction removes redundant and irrelevant features. The main purpose of this process is to reduce the training time and amount of memory required for the algorithm to work, thus reducing the computational cost when developing a predictive model (Zebari et al., 2020). In some cases, it also improves the performance of the model, although this is not always guaranteed (Benouini et al., 2020).

There are several methods available for performing feature selection in the setting of random forest classification (Speiser et al., 2019). RFs are a collection of classification and regression trees, which are simple models using binary splits on predictor variables to determine outcome predictions. Thus, they provide variable importance measures to rank predictors according to their predictive power.

#### Boruta Algorithm

Boruta is a feature selection algorithm that uses a wrapper method based on the RF classifier to measure the importance of variables. RF makes it relatively fast due to its simple heuristic feature selection procedure (Kursa, 2017).

In the Boruta algorithm, the original feature set is extended by adding shadow variables (Kursa and Rudnicki, 2010). A shadow variable is created by shuffling the values of the original feature. Several RFs are run. In each run, the set of predictor variables is doubled by adding a copy of each variable. An RF is trained on the extended data set to obtain the variable importance values. For each real variable, a statistical test is performed comparing its importance with the maximum value of all the shadow variables. If a variable systematically falls below the shadow ones, its contribution to the model is doubtful and is therefore eliminated. The shadow variables are removed, and the process continues until all variables are accepted, rejected, or a limit number of iterations is reached in which case some variables may be left undecided. This limit corresponds to the maximal number of RF runs.

In this work, we made use of the R package “Boruta” (Kursa and Rudnicki, 2020), and set the number of maximum RF to 500.

### MKL Classifier Algorithm

Kernel-based SVM employs a kernel ***k***(***x***_***i***_**,*x***_***j***_) as a function of the similarity between two instances ***x***_***i***_ and ***x***_***j***_. Given a binary classification and ***N*** labeled training instances (***x***_***i***_**,*y***_***i***_) (***y***_***i***_**ϵ*±1***) a result of training an SVM is learning the weights (α_*i*_) in the decision function:


(1) $$\begin{array}{l} \text{f}\left(\text{x}\right)=\text{sign}\left(\overset{\text{N}}{\underset{\text{i}=1}{\sum }}{\text{α}}_{\text{i}}{\text{y}}_{\text{i}}\text{k}\left({\text{x}}_{\text{i}},\;\text{x}\right)+\text{b}\right)\; \end{array}$$

The three commonly used kernels are: linear kernel **(*K***_***L***_), polynomial kernel **(*K***_***P***_), and Gaussian kernel (***K***_*G*_):


(2) $$\begin{array}{l} {\text{K}}_{\text{L}}\left({\text{x}}_{\text{i}},\;{\text{x}}_{\text{j}}\right)=\;\langle {\text{x}}_{\text{i}},\;{\text{x}}_{\text{j}}\rangle \; \end{array}$$

(3) $$K_{P}(x_{i},\;x_{j})={\left(\langle x_{i},\;x_{j}\rangle +1\right)}^{q}$$

(4) $$\begin{array}{l} K_{G}\left(x_{i},\;x_{j}\right)=\exp\left(-\frac{||x_{i}-\;x_{j}|{|}^{2}}{s^{2}}\right),\; \end{array}$$

with parameter ***q*** the polynomial degree and parameter ***s*** determine the width for Gaussian distribution.

Multiple kernel learning can be a linear or nonlinear combination of ***M*** sub-kernel functions **(**$k_{1}(x_{1}^{1},\;x)\ldots \;k_{M}(x_{1}^{M},\;x))$, where $x_{i}\;=\;{\left\{x_{i}^{m}\right\}}_{m=1}^{M}$, $x_{i}^{m}\in {\mathbb{R}}^{D_{m}}$, ***D***_***m***_ denotes the dimensionality of the ***m***^***th***^ feature representation. The methods aim to construct an optimal kernel model where the kernel is a linear combination of ***M*** fixed base kernels. Learning the kernel then consists of learning the weighting coefficients **β*=[β_1_***,**β*_2_*..**,**β*_m_***] for each base kernel, rather than optimizing the kernel parameters of a single kernel.


(5) $$\begin{array}{l} K_{opt}\left(x_{i},x_{j}\right)=\;\overset{M}{\underset{m=1}{\sum }}\beta_{m}K_{m}\left(x_{i}^{m},x_{j}^{m}\right)\;\beta \;>0,\;\overset{M}{\underset{m=1}{\sum }}\beta_{m}=1\; \end{array}$$

Plugged into the SVM decision function leads to the following decision function:


(6) $$\begin{array}{l} f\left(x\right)=sign\left(\overset{N}{\underset{i=1}{\sum }}\alpha_{i}y_{i}\left(\overset{M}{\underset{j=1}{\sum }}{\beta_{j}k}_{j}\left(x_{i},\;x\right)\right)+b\right)\; \end{array}$$

There are several MKL algorithms. We used MKL available in the SHOGUN toolbox (Sonnenburg et al., 2010). In this implementation, the kernel functions and corresponding kernel parameters are known before training, thus, only the parameters used to combine the set of kernel functions are optimized during training. The MKL learning method can help to find which kernel or combination of kernels corresponds to a better notion of similarity for the same representation of data. Nevertheless, by using inputs coming from different representations (that have different measures of similarities corresponding to different kernels), and combining them, the learning methods can find the best kernel for data representation or the combination that includes the discriminative information the data could carry. These approaches depend on the regularization chosen for the restrictions on the kernel's weights. Regularized L1-norm induces sparsity on the kernel's coefficients obtained with a considerably large fraction of zero entries focusing on the best kernels. Using the L2-norm the solution will be non-sparse, distributing the weights over all kernels (Kloft et al., 2011). Additionally, it has been demonstrated that the L2 MKL yields better performance on most of the benchmark data sets (Yu et al., 2010).

The two-step training method used here updates the combination function and the base learner parameters in an alternating manner. The algorithm was then based on wrapping linear programs around SVMs. The outer loop optimization is related to the Semi-Infinite Linear Program (SILP) that optimizes the non-smooth dual problem formulated by Sonnenburg et al. (2005, 2006). In this approach, the optimization target function follows the structural risk minimization framework and tries to minimize the sum of a regularization term that corresponds to the model complexity and an error term that correspond to the system performance. The optimization problem modeled as a SILP problem has lower computational complexity compared to those modeled with a semidefinite programming (SDP) problem and a quadratically constrained quadratic programming (QCQP) used in one-step methods.

**Figure 3:**
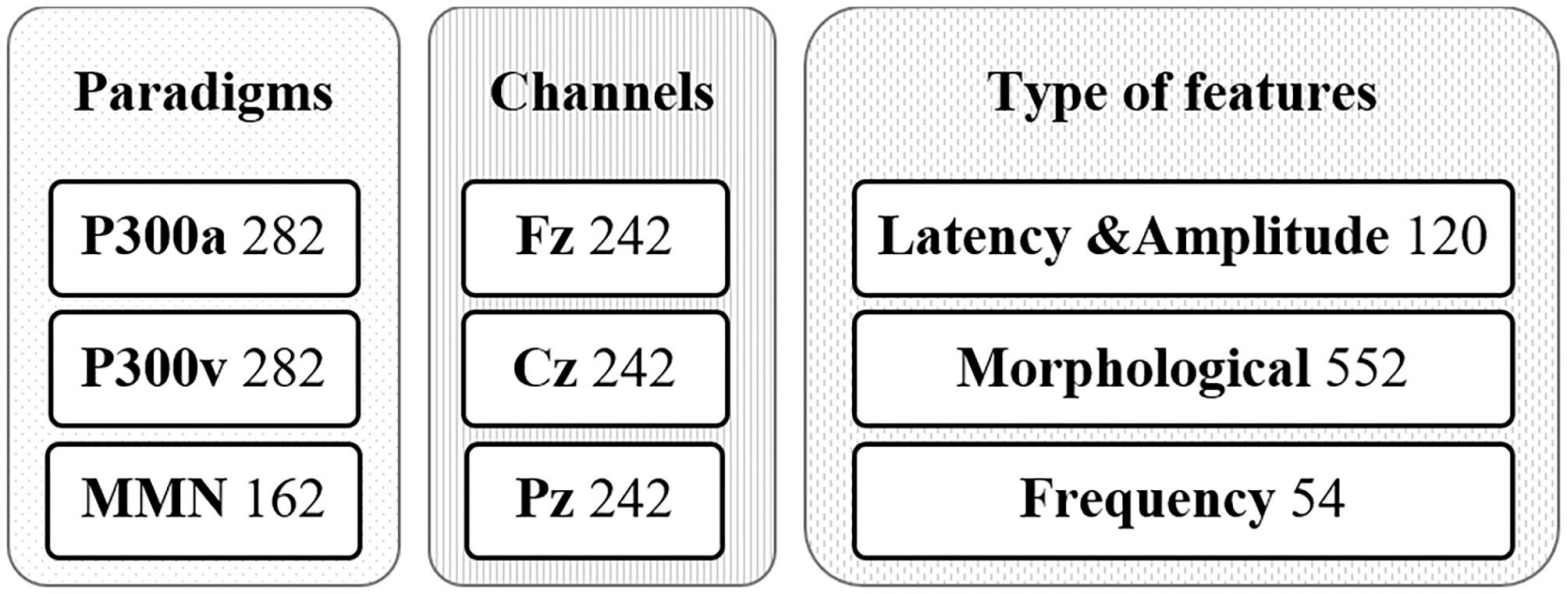
Grouping input data (726 features) in three possible kernel combinations according to the feature space approach.

In this work, the input data was mapped into different feature spaces trying to group variables with common aspects or sources: type of paradigm (P300a, P330v, MMN), channels (Fz, Cz, Pz), or type of feature (Latency & Amplitude, Morphological, Frequency) as shown in Figure 3. The 726 features were rearranged into three sources of data considering the common aspects. Thus, three different views of the data can be used to create three models to be compared. The experiments explored combinations of three kernels (one per source of data). For example, in the case of Channels the criteria used were grouping all the features from the global feature set that belong to the Cz channel and feeding a kernel to look for a notion of similarity and do the same with the other two kernels for Fz and Pz channels. The kernels were iteratively selected from a grid search of linear, polynomial, and RBF kernels with different parameters. We used a non-sparse MKL with L2-norm for thoroughly combining complementary information of the heterogeneous data sources.

### Nested Cross-Validation

**Figure 4:**
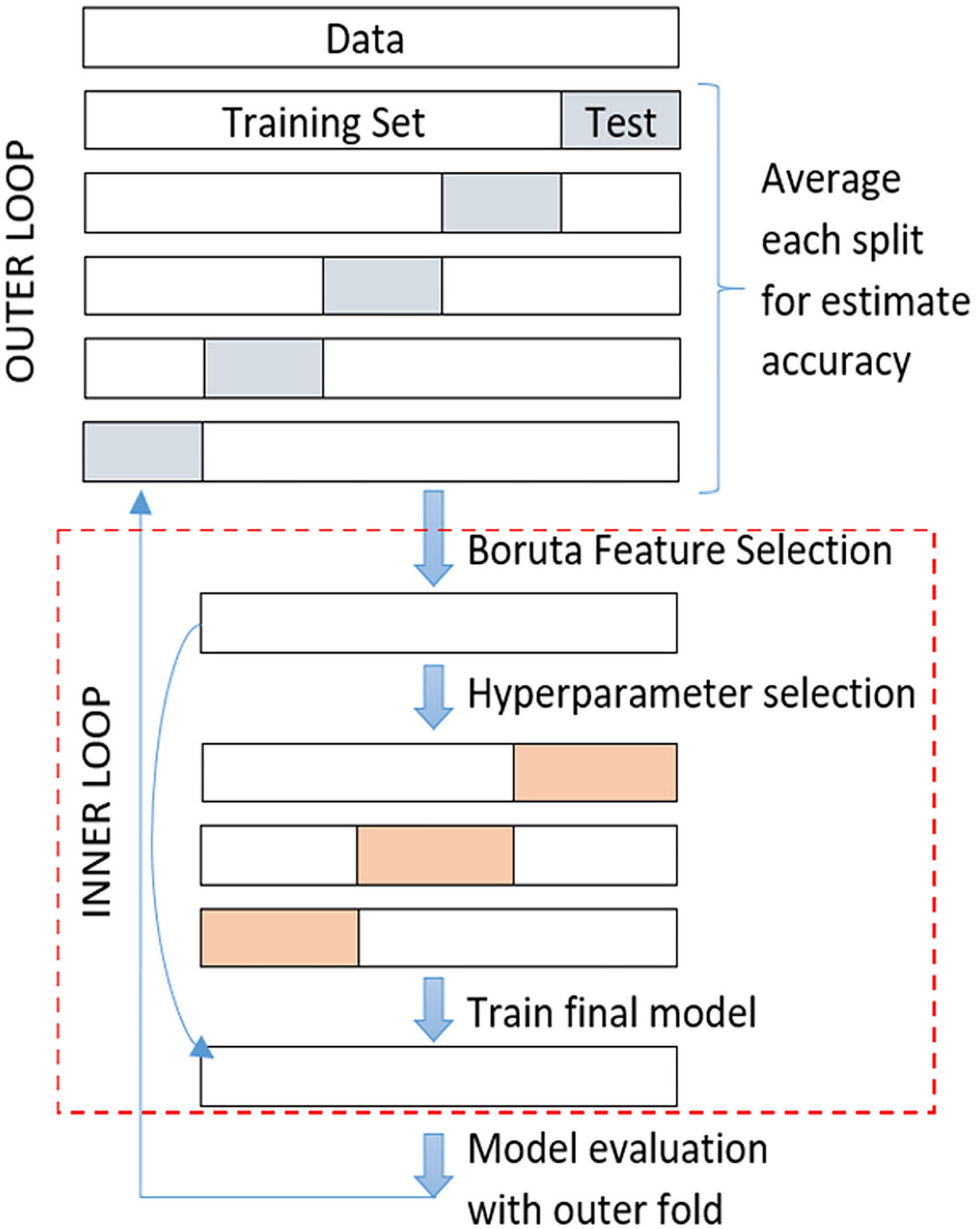
Feature selection steps applying nested cross-validation.

To explore the feature selection impact, nested cross-validation (NCV) was applied. The NCV is characterized by having an inner loop responsible for model selection/hyperparameter tuning and an outer loop is for error estimation. The entire data was divided randomly into ***k*** subsets or folds with stratification, the same proportion of patients and controls as in the complete dataset. The ***k-1*** subsets are used for feature selection and the remaining subset for testing the model after feature selection. As in the *k*-fold cross-validation method, this process was repeated ***k*** times (outer loop), each time leaving out one of the subsets reserved for testing and the rest for feature selection using the Boruta algorithm in an inner loop (see Figure 4).

Each subset obtained after feature selection was used for model hyperparameter tuning in the inner loop. One of the approaches commonly used in practice for the selection of hyperparameters is to try several combinations of them and evaluate theirs out of sample performance. The tuned parameters in the MKL classifier were:

- Regularization parameter ***C***, we evaluated C with {0.5, 1, 1.5, 5, 10}, and selected the best value considering a tradeoff between misclassification and model simplicity
- Type of kernel (linear, RBF, and polynomial)
- In the case of RBF kernels the Sigma (**σ**), we explored the following values 10, 5, 1, 0.25, 0.5, 0.75, to determine the width for Gaussian distribution.

The parameters configuration selected to train the final model was the one that reached the highest average accuracy on the inner loop. The whole dataset used for tuning parameters was then trained and tested with its corresponding test set in the outer loop. The classifiers' performance was obtained by averaging the accuracy of the ***k*** trained models.

## Results

### Feature Selection

**Figure 5:**
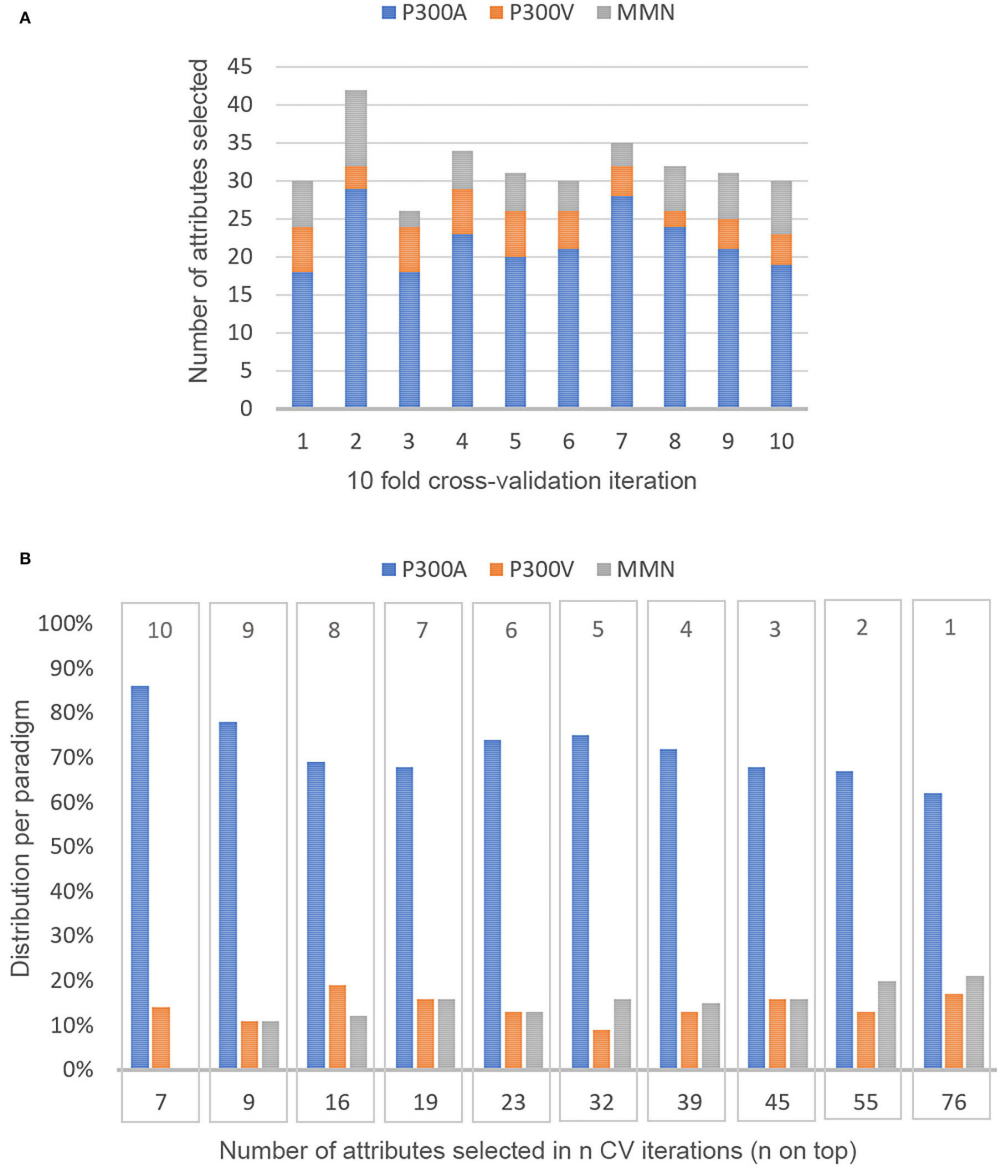
Distribution of feature selection in 10 fold cross-validation. **(A)** Amount of attributes selected per k iteration of the 10 fold CV and the distribution per paradigm in the 10 subsets of features selected, **(B)** Frequency of selection of all the attributes that were selected at least once in the ten Boruta applications. The bottom number means how many features were selected at least in *n* CV iterations (n on top).

The Boruta algorithms yielded an average of 32 (in a range of 26–42) attributes selected per ***k*** iteration in a 10-fold cross-validation (see Figure 5A). The median computation time was around 2.6 min (std 0.04), with 0.005 min per RF run. A total of 76 attributes were selected at least in one CV iteration. Figure 5B shows the number of attributes that were selected in *n* of the 10 CV iterations. The distribution of variables per paradigm is also shown. About 80% of the 76 attributes selected were related to amplitude, latency, or the correlation between them. Attributes related to the frequency domain were rarely selected. Only seven features were identified as important every time the Boruta algorithm was used. Table 3 describes these seven features according to the paradigm, type of stimulus, channel, and type of feature.

**Table 3:** Features selected by the Boruta feature selection method.

**Paradigm**	**Stimulus**	**Channel**	**Peak**	**Feature**
P300v	Target	Pz	P2	Latency
P300a	Distractor	Cz	N1	absRatio
P300a	Distractor	Fz	P2	absRatio
P300a	Distractor	Fz	P2	absAmplitude
P300a	Target	Cz	N1	absRatio
P300a	Target	Cz	N2	Latency
P300a	Target	Cz	P2	Latency

### Classifier Performance

To compare the performance of the MKL algorithms, four metrics derived from the confusion matrix were used, namely accuracy (*Acc*), area under a receiver operating characteristic (ROC) curve (*Auc*), sensibility (*Sen*) which evaluates true positive rates, and specificity (*Spe*) to evaluate the false positive rate (Kohl, 2012). The performances of the MKL classifier, with and without feature selection are summarized in Table 4.

**Table 4:** Performance of MKL algorithm with and without feature selection.

**MKL Kernels**	**Without FS**				**With FS**			
	**ACC(%)**	**SEN(%)**	**SPE(%)**	**AUC**	**ACC(%)**	**SEN(%)**	**SPE(%)**	**AUC**
Paradigm	83	80	88	0.88	86	86	87	0.92
Channels	80	74	87	0.82	84	85	86	0.91
Type of Features	82	78	85	0.87	86	86	86	0.92

## Discussion

In this work, we explored the use of MKL classification for distinguishing SZs from HCs based on ERP data. Using all features, the best classification accuracy (83%) was achieved when kernels were built by grouping features according to paradigms. Moreover, when MKL was combined with the Boruta features selection method, a classification accuracy of 86% was obtained. With this feature selection algorithm, the large number of predictor variables was reduced significantly (96%) with a lower computation time. Therefore, the training time of MKL was also reduced [0.18 (*std* 0.03) seconds per inner cross-validation loop], thus solving one of its main shortcomings: its high computational cost, especially when many features are used (de Carvalho, 2019).

The feature selection algorithm results showed that the variables that contributed most to the discrimination were related to the auditory P300 paradigm. This corresponds with the general finding that auditory P300 measures are more effective in differentiating SZs from HCs than those obtained from the visual stimuli (Park et al., 2005). The selected features were peak related, mainly related to amplitude, latency, and their combination. To a lesser extent, peak to peak related features were included in the selection. However, only three Signal related features were occasionally included. Thus, features from the frequency domain did not contribute much to the improvement of the classification.

Our results are in line with prior works. A previous study (Santos-Mayo et al., 2017) proposed a system to help diagnose schizophrenia by analyzing P300 signals during an auditory oddball task. The authors extracted time and frequency domain features similar to ours but using different collections of signals from electrodes in different regions of the scalp. Our results are comparable to theirs when the electrode groupings were used, but they obtained larger AUC values (more than 0.95) for their Left and Right hemisphere electrode groupings. We did not explore these locations. However, their dataset was unbalanced and small, which possibly limits the reliability of their findings. Other authors using also P300 amplitude and latency values as features (Shim et al., 2016) reported classification accuracies of 81% using an SVM classifier. When they combined these features with a selection of source-level density measures they increased their accuracy to 88%, a result similar to ours. Laton et al. (2014) also extracted latency and amplitude features of responses to three different odd-ball tasks and applied several classification algorithms. They achieved an average accuracy of 77% (std = 3.5). Their best result (about 85%), corresponded to an RF classifier, comparable to our results. Laton et al. (2014) also found that in a ranking of the 20 main variables contributing to the classification, 14 were extracted from the P300 auditory oddball paradigm. This suggests that, out of the three ERP paradigms used, the auditory P300 contributes most to the classification which is congruent with our findings.

One limitation of our study is that we did not use the spatial distributions of the ERPs over the scalp. Further research should include features using ERP scalp topographic maps (STM). This would take advantage of the differences in STM between schizophrenia and normal control groups reported by different authors (Morstyn et al., 1983; Frantseva et al., 2014). This is a pure image processing approach. Another track is to use independent component analysis (ICA) to split up the multi-channel ERP data into several independent spatiotemporal components. ICA separates the mixed signals into unmixed signals which are statistically independent. These approaches could generate features for a classifier. Another limitation of the present study is the small sample size which is usual in psychiatric cohorts from one site. We addressed this limitation using cross-validation strategies. However, training with larger data sets (possibly from multiple sites) would yield a more stable and reliable estimate of future performance and guarantee better generalization.

## Conclusion

Using Multiple Kernel Learning (MKL) classifiers on features defined for ERP obtained in oddball paradigms, it was possible to distinguish SZs from HCs with a classification accuracy up to 86%. Accuracy improved when the Boruta feature selection was applied. The auditory P300 provided the most informative features. Future work should explore new ERP features including topographic information.

## Data Availability Statement

The data analyzed in this study is subject to third party restrictions, which were used under license for this study. Requests to access these datasets should be directed to Laton et al. (2014).

## Ethics Statement

The provenance of data in the study (which involved human participants), and the adherence to adequate ethical standards, were reviewed and their use approved by Ethics Committee of Cuban Neuroscience Center. The patients/participants provided their written informed consent to participate in this study, as stated in the original article where they were described.

## Author Contributions

ES wrote and edited the manuscript, developed the theory, and carried out the experiments and the interpretation of the results. MO reviewed the manuscript. MV critically revised the manuscript. HS conceived the present idea and supervised it. All the authors discussed and agreed with the main focus and ideas of this article and contributed to the methodology and analysis.

## Funding

This work was supported by the VLIR-UOS project A Cuban National School of Neurotechnology for Cognitive Aging (NSNCA), Grant number CU2017TEA436A103.

## Conflict of Interest

The authors declare that the research was conducted in the absence of any commercial or financial relationships that could be construed as a potential conflict of interest.

## Publisher's Note

All claims expressed in this article are solely those of the authors and do not necessarily represent those of their affiliated organizations, or those of the publisher, the editors and the reviewers. Any product that may be evaluated in this article, or claim that may be made by its manufacturer, is not guaranteed or endorsed by the publisher.
