# Supplementary material for: Machine Learning Techniques for the Diagnosis of Schizophrenia Based on Event-Related Potentials
Source: Front Neuroinform. 2022 Jul 8;16:893788. doi: 10.3389/fninf.2022.893788 (PMC9305700; doi:10.3389/fninf.2022.893788)
Supplement: Supplementary file 1 [file Table_1.pdf]

# Supplementary Material

## 1 Annex

### Annex 1: Features definition

| | |
|---|---|
| Peak related features | |
| *Amplitude* | $A_{Peak}= max\ \{s(t), I_1 < t < I_2\}$ , Peaks P1, P3<br>$A_{Peak}= min\ \{s(t), I_1 < t < I_2\}$ , Peaks N1, N2<br>$[I_1, I_2]$ Detection Interval |
| *Latency:* | $L_{Peak}=\{t \setminus s(t) = A_{Peak}\}$ |
| *Latency/Amplitude ratio* | $LAR_{Peak}= L_{Peak}/A_{Peak}$ |
| *Absolute Amplitude* | $AA_{Peak}=\lvert A_{Peak}\rvert$ |
| *Absolute Latency/Amplitude ratio* | $ALAR_{Peak}=\lvert L_{Peak}/A_{Peak}\rvert$ |
| *Average Absolute Signal Slope* | $AASS_{Peak} = \frac{1}{n}\sum_{t=I_1}^{I_2-\tau}\frac{\lvert s(t+\tau)-s(t)\rvert}{\tau}$<br>$\tau$ is the signal sampling period, $n$ the number of samples of the digital signal |
| *Slope sign alterations* | $SSA_{Peak} = \sum_{t=I_1+\tau}^{I_2-\tau}\frac{1}{2}\lvert\frac{s(t-\tau)-s(t)}{\lvert s(t-\tau)-s(t)\rvert}+\frac{s(t+\tau)-s(t)}{\lvert s(t+\tau)-s(t)\rvert}\rvert$ |
| Peak to Peak related features | |
| *Peak to Peak* | $PP_{Peaks} = \lvert A_{Peak} - A_{NextPeak}\rvert$ |
| *Peak to Peak Time Window* | $PPT_{Peaks} = L_{NextPeak} - L_{Peak}$ |
| *Peak to Peak Slope* | $PPS_{Peaks} = PP_{Peaks}/\ PPT_{Peaks}$ |
| Signal related features | |
| *Positive Area* | $A_p = \sum_{t=-200}^{800}\frac{s(t)+\lvert s(t)\rvert}{2}$ |
| *Negative Area* | $A_n = \sum_{t=-200}^{800}\frac{s(t)-\lvert s(t)\rvert}{2}$ |
| *Total Area* | $A_{pn} = A_p + A_n$ |
| *Absolute Total Area* | $AA_{pn} = \lvert A_{pn}\rvert$ |
| *Total Absolute Area* | $AA_{pn} = A_p + \lvert A_n\rvert$ |
| *Zero Crossing* | $ZC_{Peaks} = \sum_{t=L_{Peak}}^{L_{NextPeak}}\delta_s$ , $\quad \delta_s = \begin{cases}1 & s(t) = 0\\0 & s(t) \neq 0\end{cases}$ |
| *Zero Cross density* | $ZCD_{Peaks} = \frac{ZC_{Peaks}}{PPT_{Peaks}}$ |
| *Mode frequency* | $f_{mode} = f_j,\ P_j = max\ (P_i, 1 < i < M)$ |

| | | |
|---|---|---|
| *Median frequency* | $\sum_{j=1}^{f_{median}} P_j = \sum_{j=f_{median}}^{M} P_j = \frac{1}{2}\sum_{j=1}^{M} P_j$ | $P_j$ is the power spectral density of signal at a frequency bin $j$, $M$ is the number of frequency bin in the spectrum |
| *Mean frequency* | $f_{mean} = \sum_{j=1}^{M} f_j P_j \Big/ \sum_{j=1}^{M} P_j$ | |
